# The Reducing Hospital Costs through Horizontal Integration

**Published:** 2019-11

**Authors:** Pavla Staňková, Šárka Papadaki, Ján DVORSKÝ

**Affiliations:** 1. Department of Management and Marketing, Faculty of Management and Economics, Tomas Bata University, Zlín, Czech Republic; 2. Department of Enterprise Economics, Faculty of Management and Economics, Tomas Bata University, Zlín, Czech Republic

**Keywords:** Hospitals, Costs, Horizontal integration, Effectiveness, Cost reduction

## Abstract

**Background:**

This paper investigated the impact of hospitals′ horizontal integration in the Czech Republic on the cost behavior. The aim of the research was to examined the hospitals costs in specific environment of region hospitals at NUTS 3 level (Nomenclature of Units for Territorial Statistics) – Administrative Regions.

**Methods:**

The survey was conducted in the period from April to August 2016 in the Czech Republic. The research was divided into two parts. The first part was based on data obtained from the Institute of Health Information and Statistics of the Czech Republic. We used Statgraphics Centurion XVII for the descriptive statistics and data visualization. Second part of the results was obtained through a survey research focused on managers of the horizontal integrated hospitals and their experiences with the cost behavior.

**Results:**

The results from statistical survey showed that up to 80 percent of the observed region hospitals at NUTS 3 level, the cost of treatment for a patient per day has decreased after integration into an association. Based on primary survey, 73% hospital managers confirm these results and see one of the advantages that it is possible to reduce costs through integration of hospitals. The largest savings, according to hospital managers, are due to central purchasing and investments, together and they have a better negotiation position with suppliers.

**Conclusion:**

We can confirm that horizontal integration of hospitals can lead to reduction of costs and higher efficiency, in the specific environment of region hospitals at NUTS 3 level.

## Introduction

Integrating hospitals is currently a highly-discussed matter. Most of all, its impacts on the efficiency of health care, the financial situation in hospitals, cost saving and other resulting advantages. Many authors examined the question whether the integration is really efficient. They confirm the advantages of integration and most of all highlight the following strengths of integration (1–3):

- Better placed for negotiating with suppliers
- Better condition for bulk purchases
- Increasing the quality of provided services by means of join investments into new technologies
- Reducing costs and improving professional skills by means of sharing information
- Reducing the error rate thanks to a larger number of specialized medical professionals
- Division of risks
- Join marketing strategies, etc. Some others (4, 5) present weaknesses too:
- Additional transport costs resulting from joint co-operation
- Loss of independent decision-making Integration of hospitals can happen on both vertical and horizontal scale.

a) Vertical integration

Vertical integration is based on coordinating healthcare services by complementing each other and thus fulfilling patients’ needs on various levels. Vertical integration can happen between hospital and physicians, between insurers and hospitals, between hospitals and suppliers of medicines, etc (6).

Cuellar and Gertler (7), who studied the efficiency of vertical integration on the hospital-physicians level, argue in their article that hospital–physician integration is one of the sources of the recent increase in health care costs. They find that hospital-physician integrated organizations have higher prices than stand-alone hospitals and that the differences are larger for exclusive arrangements and in less competitive markets. According to them, integrated organizations are no more efficient than stand-alone hospitals. Inconclusive results of effectiveness of vertical integration are also presented elsewhere (8). They did not find systematic significant positive effects in any form of vertical integration either. They found that an increase in the market share of hospitals with the tightest vertically integrated relationship with physicians—ownership of physician practices—was associated with higher hospital prices and spending. They found that an increase in contractual integration reduced the frequency of hospital admissions, but this effect was relatively small. Such inconclusive results are also confirmed by others. Integration leads to three evident costs, namely, monitoring, coordination, and cooperation costs (9); A greater administrative participation by physicians is consistently related to higher costs (10); Although capitation is currently having the intermediate effect of encouraging process integration, it is not yet having the ultimate anticipated effect of lowering hospital costs, etc. (11).

b) Horizontal integration

Horizontal integration is based on partnering health services which provide health services to clients on the same or similar level. Horizontal cooperation is generally more effective than vertical cooperation at improving financial performance (12). Hospital managers should consider the negative interaction effect when making decisions about whether to recommend a cooperative relationship of a horizontal or vertical direction. In addition, managers should be aware of the limited financial benefits of cooperative behavior. Dranove et al (13) presented, amongst other things, the following efficiency outcomes of horizontal integration of hospitals:

- System hospitals do not, in general, have lower patient care costs than their non-integrated counterparts.
- Integrated hospital systems are more likely than their non-integrated hospital counterparts to have unusually high administrative costs.
- Hospital systems may still be profitable if they can generate marketing benefits. Systems do not, in general, exhibit production efficiencies.

The conceptual framework for the hospitals integration in the Czech Republic was based on the privatization of the hospitals in 2003, when ownership of approximately half of the hospitals in the Czech Republic was transferred from the state to 14 newly formed, self-governing regions. The main cause of the transfer efforts was the high indebtedness of the district hospitals. Transfers of regional hospitals to business companies continued in the years to come (14). Despite the fact that the main purpose of privatization was to curb costs and increase efficiency, these stand-alone hospitals were not effectively managed that caused the hospitals horizontal integration.

The aim of this paper was to evaluate the impact of hospital′s horizontal integration in the Czech Republic on the cost behavior in specific environment of region hospitals at NUTS 3 level – Administrative regions. The Classification of Territorial Units for Statistics (NUTS) is instrumental in the European Union's Structural Fund delivery mechanisms and for locating the area where goods and services subject to European public procurement legislation are to be delivered. In the Czech Republic are presented NUTS as following: NUTS 1 – Czech Republic, NUTS 2 – Territorial regions, NUTS 3 – Administrative regions, NUTS 4 – Districts, NUTS 5 – Municipalities. The research fills a research gap by examining specific cost indicator - Proportional cost of one day of healthcare in specific environment of region hospitals at NUTS 3 level.

## Materials and Methods

The research sample included complete sample of 35 horizontal integrated hospitals owned by regions on the level NUTS 3: Health holding Královéhradecký region (5 hospitals), Health holding South Bohemia Hospitals (8 hospitals), The Ústí nad Labem region hospitals (5 hospitals), Hospital holding of the Středočeský region (5 hospitals), Health holding of the Plzeň region (6 hospitals), Hospitals of the Pardubický region (5 hospitals).

The research was divided into two parts. The first was focused on the cost effectiveness within cost of one day of healthcare. The second part of the research was the pilot survey research focused on confirmation the result that the horizontal integration is cost-effectiveness in point of view hospital managers too. We were looking for the key areas of the cost reduction from the hospital managers’ perspective.

As mentioned, the first part of the research was focused on hospital costs. For the data analysis intuitive software, data visualization and predictive analytics and Statgraphics Centurion XVII were used. It is comprehensive software. The data were collected from complete sample of 35 hospitals owned by regions on the level NUTS 3. We only used data from 22 hospitals because only this data was correct for comparison. This data is available from the Institute of Health Information and Statistics of the Czech Republic (15) and from annual reports of the hospitals. The data were categorized using the methods of grading into six regions, according to the owner. The monitored research period was from 2002 to 2015.

The aim of this statistical survey was to compare patient treatment costs before hospitals are integrated into an association and patient treatment costs after hospitals are integrated into an association. The statistical hypothesis was that we expect that the cost of treating patients per day in the hospital were lower after integration into the associations.

Cost of one day of healthcare – it is a proportional amount calculated with the aid of the annual statistic economic report of a given health care establishment and is calculated with the following formula:

Proportional cost of one day of healthcare = L*{1+(D+J+N)/(L+A)}/T

where:

L = cost of inpatient healthcare
D = cost of health care transport
J = cost of other healthcare
N = cost of non-medical services
A = cost of outpatient healthcare
T = number of days of provided healthcare

From the economic professionals’ point of view, this method is not truly exact but it provides an approximate value of the cost of one day of healthcare.

To meet the main goal of the article we used the simple classification method with a statistical criterion (hospital transformation) determined the absolute count and the relative count (percentages - %) of the hospitals in the selected region in the selected period (in the first step). In the second step, we calculated the cost of one day of patient care for each subject (hospital) using a formula (2). In the third step we used descriptive cost characteristics of the patient's treatment (CZK/day), such as mean, variance, standard deviation, lower quartile and upper quartile. The above descriptive characteristics have been identified already ( Table 1–2) as the transformation of the hospital from public to private. We used graphical analysis tools (pie chart, column graph, line graph, and cumulative bar chart) to visualize analyzed data.

**Table 1:** Patient treatment costs (CZK/day) – before integration into the association (own research)

***Variable***		***Basic statistical characteristics of the patient's costs of treatment (CZK / day)***				
***Selected Regions of the Czech Republic***	***Observed Hospital (count)***	***Mean***	***Variance***	***Standard deviation***	***Lower Quartile***	***Upper Quartile***
Central Bohemia r.	3	3 852	555 025	745	3 244	4 056
The Pardubice r.	3	3 450	463 761	681	2 806	3 905
The Pilsen r.	4	3 250	708 964	842	2 147	3 078
Hradec Králové r.	3	3 754	248 004	498	2 578	3 213
The Ústí r.	4	3 885	431 649	657	2 208	3 314
South Bohemia r.	5	3 571	505 521	711	3 010	3 844

**Table 2:** Patient treatment costs (CZK/day) – after integration into the association (own research)

***Variable***		***Basic statistical characteristics of the patient's costs of treatment (CZK / day)***				
***Selected Regions of the Czech Republic***	***Observed Hospital (count)***	***Mean***	***Variance***	***Standard deviation***	***Lower Quartile***	***Upper Quartile***
Central Bohemia r.	3	3 685	474 721	689	3 105	3 784
The Pardubice r.	3	3 581	344 569	587	2 741	3 554
The Pilsen r.	4	3 471	218 089	467	2 297	3 361
Hradec Králové r.	3	3 317	289 444	538	2 947	3 125
The Ústí r.	4	3 489	729 314	854	2 108	3 231
South Bohemia r.	5	3 225	506 944	712	2 874	3 647

The data from annual reports of the hospital were not consistent. The process of preparing annual reports in hospitals is not uniform in the Czech Republic. We used data from 62.9% (22/35) horizontal integrated hospitals owned by regions on the level NUTS 3 because there were not found data in each year of the monitored period of other hospitals (12 hospitals). Major flaws of other hospitals: problem of hospital (inability to publicize data) and problem of state organizations (hospitals aren′t sanctioned for failure to publicize data). By comparing the results of quantitative data analysis (2004–2015) and questionnaire survey of hospital managers (2016), the reliability and relevance of research were improved. The pilot study was based on primary research through a questionnaire. The questionnaire included 19 questions; most of the questions were constructed as closed. In total, it was sent via e-mail to all 35 directors of research sample hospitals which are part of holding companies or other forms of associations. Of this number of respondents, we received 15 responses; the response rate was approximately 43%. It was a very nice response with a good return rate, in the Czech conditions (the common return rate is from 7 to 8 %). The survey was conducted in the period from April to August 2016. The first phase consisted of a pre-test questionnaire. The second phase was the undertaken research itself. A questionnaire focused on general hospital associations and their advantages and disadvantages, one individual area was cost management. Part of this article is to evaluate only the selected questions, which concern the influence of associations on costs.

## Results

### Results of the statistical survey

We performed descriptive statistics which were calculated from the basic statistical characteristics of the patient's treatment costs per day of admission in hospital. These statistical characteristics are: mean, variance, standard deviation, lower quartile, upper quartile. Interpretation for example “the mean” is the costs of admission of a patient in one hospital (CZK/day) in the selected hospitals owned by NUTS 3 regions of the Czech Republic. The results of correlation matrix (Table 3) between horizontal integration of hospital (HIH) and reducing hospital cost (RHC) showed very strong dependency (R > 0.9). The results of the descriptive statistics are summarized in Table 1 and 2.

**Table 3:** Correlation matrix between horizontal integration of hospital (HIH) and reducing hospital cost (RHC) (own research)

***Variable***	***HIH***	***RHC***
HIH	1	0.9764
RHC	0.9764	1

The lower quartile can be interpreted as 25 percent of all costs (per day) in the association owned by NUTS 3 region is lower than 3 244 CZK. The upper quartile can be interpreted as 25 percent of all costs (per day) in the region association is higher than 4 056 CZK. The difference between the lower quartile and the upper quartile can be interpreted as 50% of all collected data are in this interval. For example: Patient treatment costs before integration into the Association were within 3 244 CZK to 4 056 CZK per day in the Central Bohemia Regional Hospital. If variability increases in the interval between the lower and upper quartile then costs are more inhomogeneous.

**Fig. 1:**
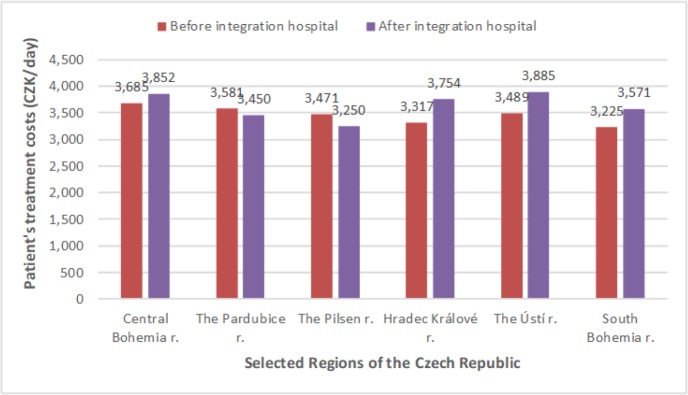
Comparison mean costs of patient's treatment (own research)

Fig. 1 shows a comparison of patient treatment costs before integration into the association and patient treatment costs after integration into the association. The grey columns are the mean patient treatment cost per day before integration into the association; the black columns are the mean patient treatment cost per day after integration into the association.

In up to 80% of the observed hospitals the cost of treatment for a patient per day has decreased. Patient treatment costs decreased by an average of 166 CZK per day. The largest financial savings after integration into the association were in the hospitals owned by the Hradec Kralove Region.

Rising financial funds after integration into the associate hospital were in the hospitals owned by the Pardubice region and the Pilsen region. Numerically calculated descriptive statistics by using basic statistical characteristics and graphic analysis of data by using a bar chart confirmed the statistical hypothesis that costs of patient treatment were reduced after integration into an association.

### Results of the questionnaire survey

**Fig. 2:**
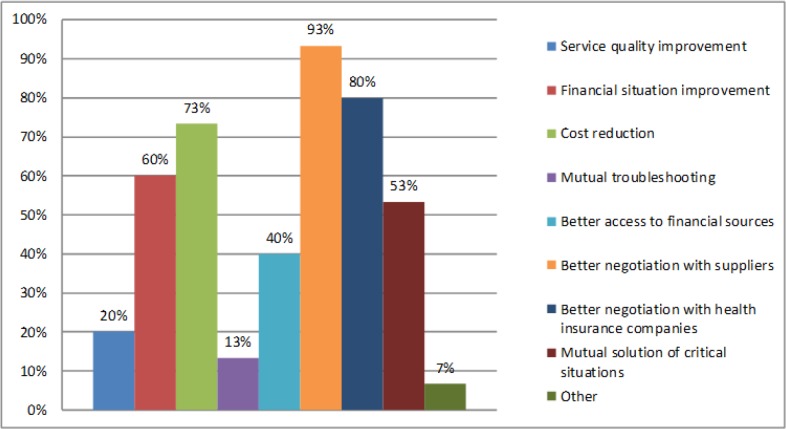
The main advantages of horizontal integration of hospitals (own research)

In this second part we will focus on the results of the questionnaire survey. The first question relates to the benefits of an association of hospitals. Here we are interested in whether directors see, as one of the benefits of an association of hospitals, a reduction in costs. From the results, we see that 73% of respondents see one of the advantages to be a reduction in the costs at individual hospitals (Fig. 2). We confirmed the first part research results that the horizontal integration in the specific environment of region hospitals at NUTS 3 level can lead to cost reduction.

**Fig. 3:**
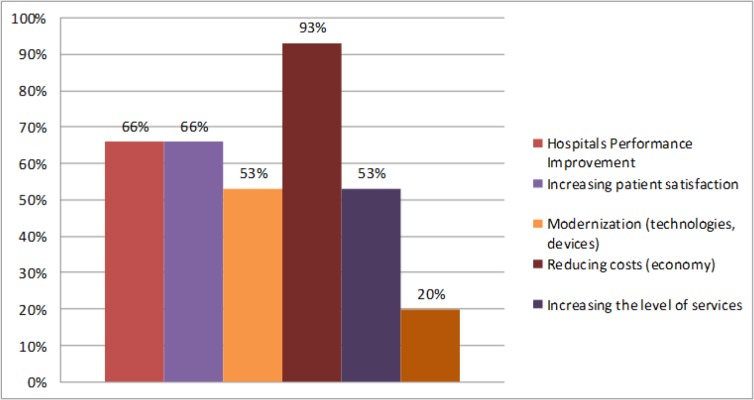
Topics of joint projects (own research)

Another question concerned the project themes which are dealt with under associated hospitals. As many as 90% of all projects relate to cost management and cost reduction. Hospitals in the holding undertake projects related to cost reduction and efficiency. Topics of other joint projects are in Fig. 3.

**Fig. 4:**
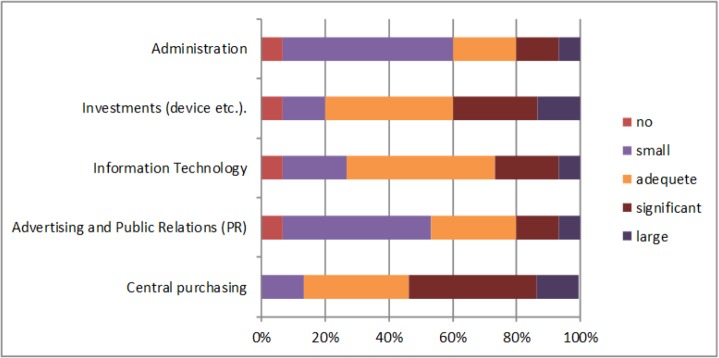
Areas of cost savings (own research)

**Fig. 5:**
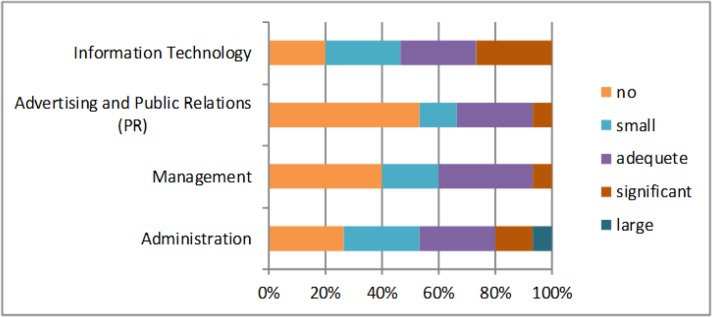
Areas of costs growing (own research)

The next question was about the areas of cost saving, the largest saving is made in the area of central purchasing and also in investments (Fig. 4). So hospitals save the largest amount of money in central purchasing and investments, which mean that they buy materials, equipment, medicaments etc. together and they have a better negotiation position with suppliers. The hospitals can see that in some areas costs could be growing. The largest cost growth is in two areas, in administration and in information technology. In the other areas costs are not growing significantly (Fig. 5).

## Discussion

By comparing the cost per day of treating a patient in the merged hospitals before and after the change, we can confirm that the merger of hospitals leads to cost reduction similar to others (12, 16). One of the benefit was lowering costs. The other benefits were eliminating unneeded services, economics of scale, increased market and negotiating power, profit and market share gains, better recruitment and longer retention of staff and also environmental acceptance. Efficiency gains are possible through horizontal integration of hospitals (17).

The views of managers of hospitals also support these results. According to our research we can say that the managers of hospitals see as one of the biggest advantages in holdings is cost reduction (73% of respondents). The managers confirm the following main advantages of horizontal integration: better negotiation with suppliers (93% of respondents), better negotiation with health insurance companies (80% of respondents), financial situation improvement (60% of respondents) and mutual solution of critical situation (53% of respondents). Most hospitals in holdings save in central purchases and joint investments. Conversely, respondents see an increase in the cost of administration and information technology.

These results were confirmed in the conference about healthcare effectiveness in Prague. There were confirms on the cost reduction, especially the cost related to the joint purchasing of medicines, materials and services, the costs associated with personnel and wage policy and internal costs of outsourcing. There were discussed the main problems associated with the hospital horizontal integration process, too. According to the experience of managers it was very difficult, in particular, the integration process required high standards on the management of merger and restructuring, it was necessary to reinforce the economy of the hospital by debiting or investing property due to the long-term poor economic situation of the hospital in some cases. Problems of the integration process were also caused by inconsistent process management of hospitals.

From the research results we can utilize the specific knowledge essential for effective management of horizontally integrated hospitals:

- Horizontal integrated hospitals must be managed as commercial firms whose cost management is a necessary part of the hospital management,
- Cost management common to all hospitals in the holding must be regulated by uniform procedures and guidelines,
- Central purchasing is cost-effective,
- Horizontal integration brings greater negotiating power to suppliers, which is positively reflected in costs,
- Cost savings also result from joint negotiations with health insurers,
- Cost-effective is the centralization and sharing of services.

## Conclusion

This study sought to examine the impact of horizontal integration on cost-effectiveness in the specific environment of region hospitals at NUTS 3 level. The presented research results confirmed that the horizontal integration lead to cost savings and higher efficiency, regardless the environment conditions, including the specific environment of region hospitals at NUTS 3 level.

## Ethical considerations

Ethical issues (Including plagiarism, informed consent, misconduct, data fabrication and/or falsification, double publication and/or submission, redundancy, etc.) have been completely observed by the authors.
